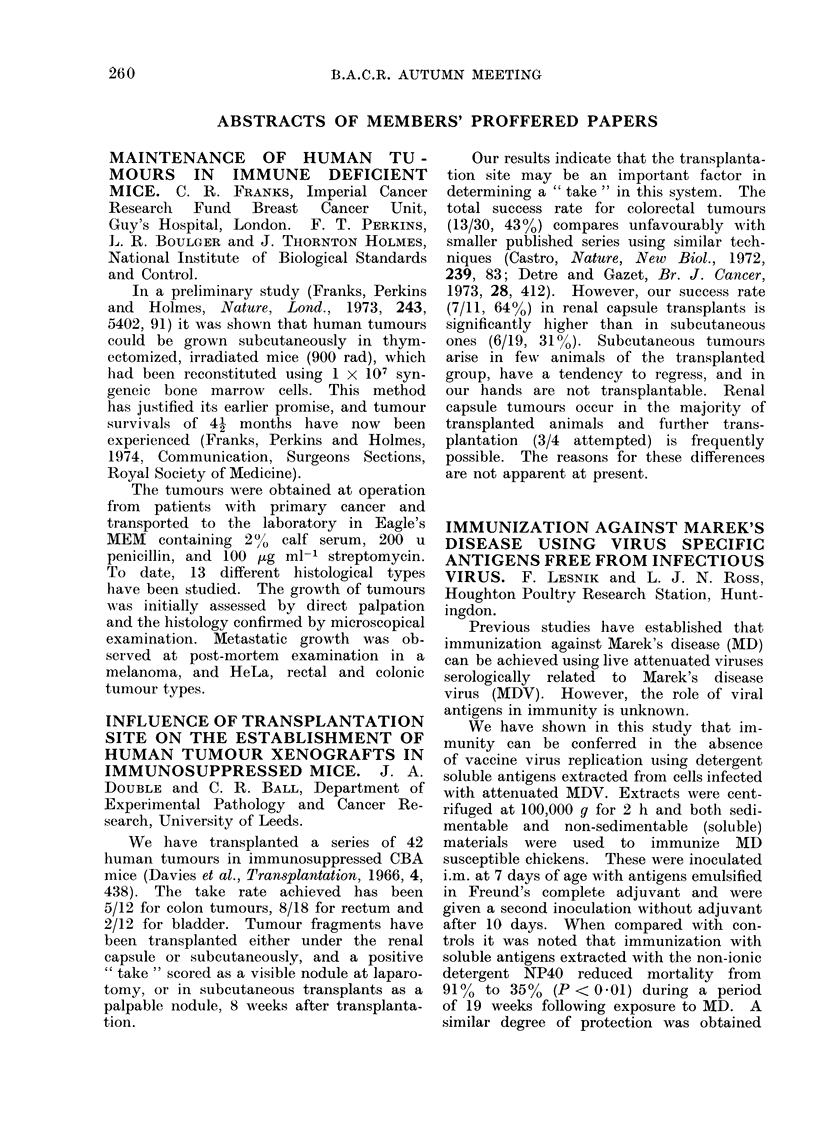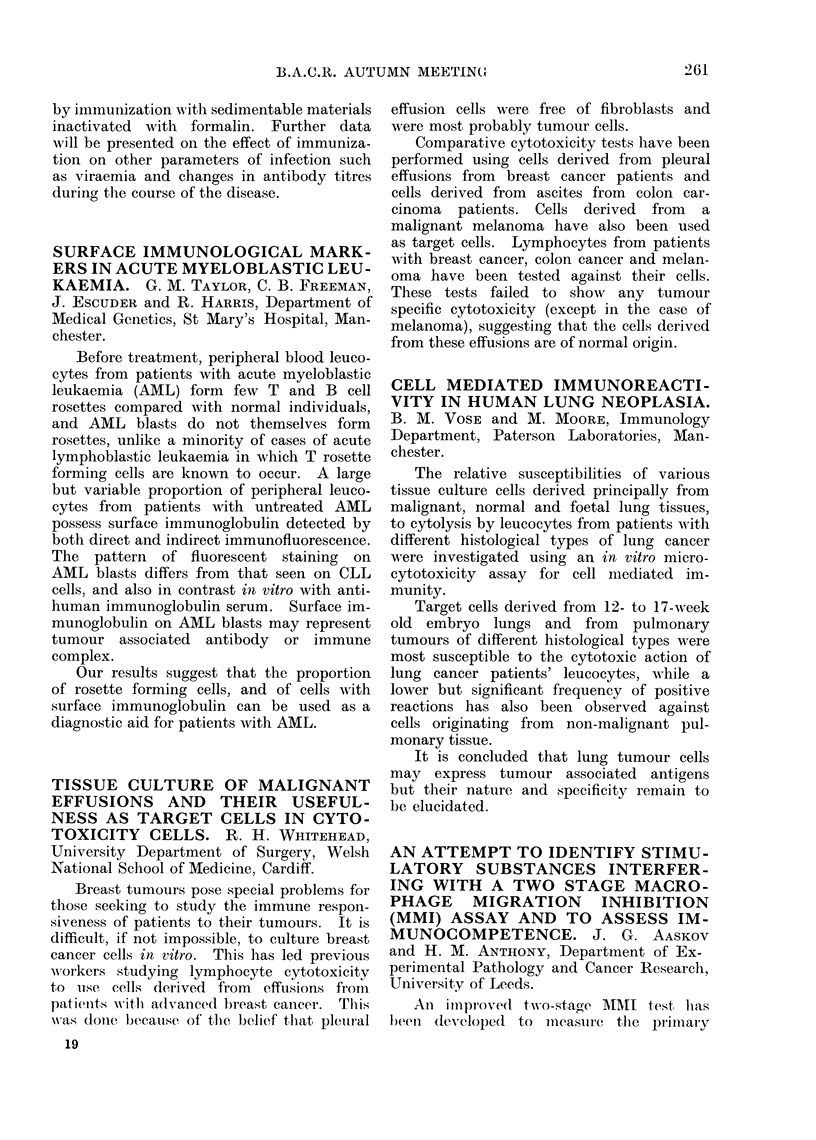# Proceedings: Immunization against Marek's disease using virus specific antigens free from infectious virus.

**DOI:** 10.1038/bjc.1975.38

**Published:** 1975-02

**Authors:** F. Lesnik, L. J. Ross


**IMMUNIZATION AGAINST MAREK'S DISEASE USING VIRUS SPECIFIC ANTIGENS FREE FROM INFECTIOUS VIRUS.** F. LESNIK and L. J. N. ROSS, Houghton Poultry Research Station, Huntingdon.

Previous studies have established that immunization against Marek's disease (MD) can be achieved using live attenuated viruses serologically related to Marek's disease virus (MDV). However, the role of viral antigens in immunity is unknown.

We have shown in this study that immunity can be conferred in the absence of vaccine virus replication using detergent soluble antigens extracted from cells infected with attenuated MDV. Extracts were centrifuged at 100,000 *g* for 2 h and both sedimentable and non-sedimentable (soluble) materials were used to immunize MD susceptible chickens. These were inoculated i.m. at 7 days of age with antigens emulsified in Freund's complete adjuvant and were given a second inoculation without adjuvant after 10 days. When compared with controls it was noted that immunization with soluble antigens extracted with the non-ionic detergent NP40 reduced mortality from 91% to 35% ($P < 0{\cdot}01$) during a period of 19 weeks following exposure to MD. A similar degree of protection was obtained by immunization with sedimentable materials inactivated with formalin. Further data will be presented on the effect of immunization on other parameters of infection such as viraemia and changes in antibody titres during the course of the disease.